# Cardiopulmonary Resuscitation Preferences of People Receiving Dialysis

**DOI:** 10.1001/jamanetworkopen.2020.10398

**Published:** 2020-08-24

**Authors:** Gwen M. Bernacki, Ruth A. Engelberg, J. Randall Curtis, Manjula Kurella Tamura, Lyndia C. Brumback, Danielle C. Lavallee, Elizabeth K. Vig, Ann M. O’Hare

**Affiliations:** 1 Department of Medicine, University of Washington, Seattle; 2 Cambia Palliative Care Center of Excellence, University of Washington, Seattle; 3 Department of Medicine, Stanford University Medical Center, Palo Alto, California; 4 Division of Nephrology, Geriatric Research, Education and Clinical Center, VA Palo Alto Health Care System, Palo Alto, California; 5 Department of Biostatistics, University of Washington, Seattle; 6 Department of Surgery, University of Washington, Seattle; 7 Hospital and Specialty Medicine, VA Puget Sound Health Care System, Seattle, Washington; 8 Geriatrics, VA Puget Sound Health Care System, Seattle, Washington; 9 Kidney Research Institute, University of Washington, Seattle

## Abstract

**Importance:**

Whether the cardiopulmonary resuscitation (CPR) preferences of patients receiving dialysis align with their values and other aspects of end-of-life care is not known.

**Objective:**

To describe the CPR preferences of patients receiving dialysis and how these preferences are associated with their responses to questions about other aspects of end-of-life care.

**Design, Setting, and Participants:**

Cross-sectional survey study of a consecutive sample of patients receiving dialysis at 31 nonprofit dialysis facilities in 2 US metropolitan areas (Seattle, Washington, and Nashville, Tennessee) between April 22, 2015, and October 2, 2018. Analyses for this article were conducted between December 2018 and April 2020.

**Exposures:**

Participants were asked to respond to the question “If you had to decide right now, would you want CPR if your heart were to stop beating?” Those who indicated they would probably or definitely want CPR were categorized as preferring CPR.

**Main Outcomes and Measures:**

This study examined the association between preference for CPR and other treatment preferences, engagement in advance care planning, values, desired place of death, expectations about prognosis, symptoms, and palliative care needs.

**Results:**

Of the 1434 individuals invited to complete the survey, 1009 agreed to participate, and 876 were included in the analytic cohort (61.1%). The final cohort had a mean (SD) age of 62.6 (14.0) years; 492 (56.2%) were men, and 528 (60.3%) were White individuals. Among 738 of 876 participants (84.2%) who indicated that they would definitely or probably want CPR (CPR group), 555 (75.2%) wanted mechanical ventilation vs 13 of 138 (9.4%) of those who did not want CPR (do not resuscitate [DNR] group) (*P* < .001). A total of 249 of 738 participants (33.7%) in the CPR group vs 84 of 138 (60.9%) in the DNR group had documented treatment preferences (*P* < .001). In terms of values about future care, 171 participants (23.2%) in the CPR group vs 5 of 138 (3.6%) in the DNR group valued life prolongation (*P* < .001); 320 in the CPR group (43.4%) vs 109 of 138 in the DNR group (79.0%) valued comfort (*P* < .001); and 247 participants (33.5%) in the CPR group vs 24 of 138 (17.4%) in the DNR group were unsure about their wishes for future care (*P* < .001). In the CPR group, 207 (28.0%) had thought about stopping dialysis vs 62 of 138 (44.9%) in the DNR group (*P* < .001), and 181 (24.5%) vs 58 of 138 (42.0%) had discussed stopping dialysis (*P* = .001). No statistically significant associations were observed between CPR preference and documentation of a surrogate decision maker, thoughts or discussion of hospice, preferred place of death, expectations about prognosis, reported symptoms, or palliative care needs.

**Conclusions and Relevance:**

The CPR preferences of patients receiving dialysis were associated with some, but not all, other aspects of end-of-life care. How participants responded to questions about these other aspects of end-of-life care were not always aligned with their CPR preference. More work is needed to integrate discussions about code status with bigger picture conversations about patients’ values, goals, and preferences for end-of-life care.

## Key Points

**Question:**

Do the cardiopulmonary resuscitation (CPR) preferences of patients receiving dialysis align with their responses to questions about other aspects of end-of-life care?

**Findings:**

Among 876 participants in this cross-sectional survey study, most (84.2%) indicated they would definitely or probably want CPR. Preference for CPR was associated with some, but not all, of the other domains of end-of-life care that were examined; responses to questions about these other aspects of end-of-life care were not always aligned with participants’ CPR preference.

**Meaning:**

Code status discussions with patients receiving dialysis should be integrated with broader conversations about their values, goals, and preferences for end-of-life care.

## Teaser

This cross-sectional survey study describes the cardiopulmonary resuscitation preferences of patients receiving dialysis and how these preferences are associated with their responses to questions about other aspects of end-of-life care.

## Introduction

People with end-stage kidney disease (ESKD) receiving maintenance dialysis have mortality rates of 15% to 20% per year.^1^ Compared with the general population, these patients are far more likely to die prematurely of a cardiac arrest.^2,3,4,5^ Out-of-hospital sudden cardiac death occurs 20 times more often in people receiving dialysis than in the general population.^1,2,3,6^ Less than 25% of patients who experience a cardiac arrest during dialysis treatment survive to hospital discharge, and only 8% to 15% survive more than 1 year after the arrest.^3,6,7,8,9,10^ Most prior studies have also shown that outcomes after in-hospital cardiac arrest are less favorable for these patients than for more broadly defined populations, with only one-half surviving beyond 5 months (compared with almost 3 years for the general population).^11,12^ Patients receiving maintenance dialysis who survive a cardiac arrest in the hospital are also more likely to be discharged to a skilled nursing facility compared with other survivors of cardiac arrest.^13^

Despite their generally poor outcomes after resuscitation, available data suggest that patients receiving dialysis are much more likely to receive cardiopulmonary resuscitation (CPR) than members of the general population. These patients are also more likely to receive intensive patterns of end-of-life care focused on life prolongation compared with some other seriously ill populations.^11,14,15,16,17^ The extent to which these aggressive patterns of end-of-life care focused on life extension align with the values, goals, and preferences of individual patients receiving dialysis is not known.^18^ Although most patients with advanced kidney disease say that they would want to be resuscitated,^19,20,21^ most also indicate that they would value comfort and relief of suffering if they were seriously ill or dying and that they would prefer to die at home.^20^

Because resuscitation preferences are often elicited reflexively at times of transition, such as hospital admission or dialysis initiation or when obtaining consent for procedures, these conversations may not be integrated with a broader process of advance care planning that explores patients’ values, goals, expectations, and preferences for future care.^22,23^ Although at least 1 study^3^ has attempted to gauge the extent to which the resuscitation preferences of patients undergoing dialysis are honored, no prior studies to our knowledge have evaluated the association between patients’ resuscitation preferences and how they respond to questions about other aspects of end-of-life care. This study used data collected from patients receiving dialysis who participated in a survey about end-of-life care to describe the association of resuscitation preferences with self-reported participant characteristics and with other domains of palliative and end-of-life care.

## Methods

### Study Design

In this cross-sectional survey study, we analyzed data from the United States Renal Data System Study of Treatment Preferences (USTATE) conducted among patients at 31 nonprofit dialysis facilities in 2 US metropolitan areas (Seattle, Washington, and Nashville, Tennessee) between April 22, 2015, and October 2, 2018, in accordance with best practices for survey research.^24,25^ Analyses for this article were conducted between December 2018 and April 2020. Patients were eligible to participate in USTATE if they were 21 years or older, sufficiently fluent to complete surveys in English, cognitively able to provide written informed consent, and receiving maintenance dialysis. Study staff consulted with dialysis facility charge nurses to identify patients who met these eligibility criteria and then approached eligible patients in person during their dialysis session, resulting in a pragmatic consecutive sample of eligible patients receiving dialysis at each facility at the time of survey administration. Most patients approached were receiving in-center hemodialysis; a small convenience sample of patients were receiving peritoneal dialysis. Study participants could choose whether to complete the survey themselves or have a study coordinator record their responses to survey questions during their dialysis session. The study was approved by the institutional review board at the University of Washington, Seattle, and written informed consent was obtained from participants. This study followed the Strengthening the Reporting of Observational Studies in Epidemiology (STROBE) reporting guideline.

### Survey

The USTATE survey included items that had been previously validated as well as items developed by the study investigators (R.A.E., M.K.T., D.C.L., E.K.V., and A.M.O.) that were refined during the pilot testing phase of the survey.^20,24,26,27,28,29,30,31^ The survey included questions about patients’ preference for CPR as well as about their values, preferences, knowledge, and expectations pertaining to other aspects of end-of-life care, including self-reported engagement in advance care planning, values around life prolongation, knowledge and preferences about other care decisions (ie, mechanical ventilation, dialysis discontinuation, and hospice enrollment), preferred place of death, expectations about prognosis, symptom burden, and palliative care needs (eAppendix in the Supplement). Respondents were also asked to provide some basic information about themselves (eg, age, sex, race, ethnicity, highest educational level, importance of spiritual and/or religious beliefs, time receiving dialysis, and self-reported health assessment).

### Primary Exposure

Responses to the following question were used to define the primary exposure: “If you had to decide right now, would you want CPR (cardiopulmonary resuscitation) if your heart were to stop beating?” Possible responses included definitely yes, probably yes, probably no, and definitely no. For the analyses described herein, participants who indicated they would probably or definitely want CPR were classified as preferring CPR, whereas those who said they would probably or definitely not want CPR were classified as preferring not to be resuscitated (do not resuscitate [DNR]).^32^

### Outcomes

This study examined the association between preference for CPR and 9 study outcomes. Outcome 1 was the preference for receipt of mechanical ventilation; participants were asked if they would want this intervention if they “had to decide right now,” and those indicating they would definitely or probably want this intervention were classified as preferring mechanical ventilation. Outcome 2 was self-reported engagement in advance care planning, which included (1) documentation of a surrogate decision maker (“I have signed official papers naming someone to make medical decisions for me [eg, as part of a living will or advance directive]” and have or have not discussed this choice with him or her) and (2) documentation of treatment preferences (“I have signed official papers documenting my preferences (eg, living will or advance directive)” and have or have not talked with at least one friend or family member about this preference). Outcome 3 was the participant’s values around life prolongation. Participants were queried about their preferred plan of care if they “were to become very sick in the future,” with responses categorized as “extending life even if that means having more pain and discomfort” vs “relieving pain and discomfort as much as possible, even if that means not living as long” vs “I’m not sure which I would choose”. Outcome 4 was whether participants had had thoughts or prior discussions about stopping dialysis vs not. Outcome 5 was whether they had had thoughts or prior discussions about receiving hospice care if they were to become sicker or if their goals changed vs not. Outcome 6 was the respondent’s desired place of death (categorized as home if home or the home of a relative or friend or as other if hospital, nursing home, or other setting). Outcome 7 was the expectation about prognosis (categorized as predicted life expectancy of <5 years vs 5-10 years vs >10 years vs unsure). Outcome 8 was the presence or absence of symptoms over the past week, including (1) weakness or lack of energy, (2) pain, (3) difficulty sleeping, (4) poor mobility, (5) anxiety, (6) shortness of breath, and (7) depression. Outcome 9 was the presence of palliative care needs, including (1) “would like someone to talk to about treatment options for the future” vs not, (2) “would like help with making plans in case I become very ill (advance care planning)” vs not, (3) “would like someone to talk to about my care plan and treatments” vs not, (4) “would like to have someone to talk to about finding meaning in my life now” vs not, (5) “would like help with learning to cope with feelings of sadness” vs not, and (6) “would like to have someone to talk to about dying and death” vs not.

### Covariates

Multivariable analyses were adjusted for self-reported participant characteristics. These included age (categorized as <60, 60-74, and ≥75 years), sex, race (categorized as White, Black, or other, which included Asian, American Indian, Alaskan Native, Native Hawaiian, or other Pacific Islander), ethnicity (categorized as non-Hispanic vs Hispanic), highest educational level (categorized as no college vs at least some college/trade school), importance of spiritual and/or religious beliefs based on responses to the statement “My religious and spiritual beliefs are really what lie behind my whole approach to life” (categorized as definitely true or tends to be true vs tends not to be true or definitely not true), length of time receiving dialysis (categorized as <1 year, 1-5 years, or >5 years), and self-reported health status (categorized as good, very good, or excellent vs fair or poor) (eAppendix in the Supplement).

### Statistical Analysis

Logistic regression was used to evaluate the association of the aforementioned self-reported participant characteristics with a preference for CPR. To evaluate the association between preference for CPR and the 9 study outcomes, this study used logistic or multinomial regression depending on whether the outcome was binary or multicategorical, after adjustment for self-reported participant characteristics. Estimates of association are presented as adjusted risk differences with 95% CIs. Predicted risks of the relevant outcome for the exposure group were calculated by fixing the value of the other covariates at the mean value for the cohort. To account for multiple comparisons, a threshold of 2-sided *P* ≤ .001 was used for analyses of study outcomes. All analyses were conducted using Stata, version 13.1 (StataCorp LLC).

## Results

Between April 22, 2015, and October 2, 2018, a total of 1595 patients were approached to participate in the study. During the first 8 months of recruitment, 161 eligible patients were invited to participate in the pilot phase of the study, and 146 of these completed pilot versions of the survey (90.7% response rate). After the pilot phase, a further 1434 eligible patients were invited to participate, and 1009 patients completed the final version of the survey. Nine of these respondents were excluded because they did not record their name and/or date of birth on the paper survey or consent form, along with an additional 124 participants who did not complete one or more survey questions included in the analyses described herein, yielding an analytic cohort of 876 participants (61.1% of 1434 eligible patients invited to complete the final version of the survey).

### Cohort Characteristics and CPR Preference

Among 876 participants included in the analytic sample, the mean (SD) age at the time of survey completion was 62.6 (14.0) years; 492 (56.2%) were men, and 528 (60.3%) were White individuals. In the total sample, 248 participants (28.3%) had been receiving dialysis for less than 1 year at the time of survey administration, 429 (49.0%) had been receiving dialysis between 1 and 5 years, and 199 (22.7%) had been receiving dialysis for more than 5 years. Overall, 508 (58.0%) participants described themselves as being in good, very good, or excellent health, with 274 (31.3%) in fair health and 94 (10.7%) in poor health (Table 1).

**Table 1.** Baseline Characteristics of the Analytic Cohort

Self-reported characteristic	No. (%)^a^		
	Total sample (N = 876)	Resuscitation preference	
		Yes/CPR (n = 738)^b^	No/DNR (n = 138)^c^
Age, y			
<60	360 (41.1)	341 (46.2)	19 (13.8)
60-74	348 (39.7)	277 (37.5)	71 (51.4)
≥75	168 (19.2)	120 (16.3)	48 (34.8)
Sex			
Female	384 (43.8)	320 (43.4)	64 (46.4)
Male	492 (56.2)	418 (56.6)	74 (53.6)
Race			
White	528 (60.3)	429 (58.1)	99 (71.7)
Black	229 (26.1)	212 (28.7)	17 (12.3)
Asian, American Indian, Alaskan Native, Native Hawaiian, or other Pacific Islander	119 (13.6)	97 (13.1)	22 (15.9)
Ethnicity			
Non-Hispanic	824 (94.1)	695 (94.2)	>127 (>92.0)^d^
Hispanic	52 (5.9)	43 (5.8)	<11 (<8.0)^d^
Highest educational level			
Completed high school/GED or less	396 (45.2)	332 (45.0)	64 (46.4)
Completed at least some college/trade school	480 (54.8)	406 (55.0)	74 (53.6)
Spiritual and/or religious beliefs important			
Definitely true or tends to be true	624 (71.2)	536 (72.6)	88 (63.8)
Tends not to be true or definitely not true	252 (28.8)	202 (27.4)	50 (36.2)
Time receiving dialysis, y			
<1	248 (28.3)	211 (28.6)	37 (26.8)
1-5	429 (49.0)	348 (47.2)	81 (58.7)
>5	199 (22.7)	179 (24.3)	20 (14.5)
Self-reported health assessment			
Good, very good, or excellent	508 (58.0)	433 (58.7)	75 (54.3)
Fair	274 (31.3)	231 (31.3)	43 (31.2)
Poor	94 (10.7)	74 (10.0)	20 (14.5)

Abbreviations: CPR, cardiopulmonary resuscitation; DNR, do not resuscitate; GED, General Educational Development test.

^a^ Percentages have been rounded and may not total 100.

^b^ Preference for CPR was “probably or definitely wanting CPR.”

^c^ Preference for CPR was “probably or definitely not wanting CPR.”

^d^ Number not specified to protect confidentiality.

Among 876 participants, 572 (65.3%) said that they definitely wanted to be resuscitated and 166 (18.9%) said that they probably wanted to be resuscitated. A total of 138 participants did not prefer resuscitation; 61 (7.0%) said that they probably did not want to be resuscitated, and 77 (8.8%) said that they definitely did not want to be resuscitated.

### Association Between Self-reported Participant Characteristics and Preference for CPR

Self-reported participant characteristics that were independently associated with a preference for CPR included younger age, Black race, reporting that spiritual and/or religious beliefs were important to them, and receiving dialysis for longer periods. These results are summarized in Table 2.

**Table 2.** Association Between Self-reported Participant Characteristics and Preference for CPR

Self-reported characteristic	Adjusted risk difference (95% CI)^**a**^	*P* value
Age, y		<.001^b^
<60	1 [Reference]	NA
60-74	−15.4 (−20.3 to −10.6)	<.001
≥75	−22.9 (−30.2 to −5.6)	<.001
Sex		
Female	1 [Reference]	NA
Male	1.9 (−2.8 to 6.7)	.43
Race		
White	1 [Reference]	NA
Black	6.3 (0.8 to 11.8)	.03
Asian, American Indian, Alaskan Native, Native Hawaiian, or other Pacific Islander	4.5 (−12.4 to 3.5)	.27
Ethnicity		
Non-Hispanic	1 [Reference]	NA
Hispanic	5.7 (−6.2 to 17.6)	.35
Highest educational level		
Completed high school/GED or less	1 [Reference]	NA
Completed at least some college/trade school	3.1 (−1.7 to 7.9)	.21
Spiritual and/or religious beliefs important		
Definitely true or tends to be true	1 [Reference]	NA
Tends not to be true or definitely not true	−6.5 (−12.1 to 0.9)	.02
Time receiving dialysis, y		.04^b^
<1	1 [Reference]	NA
1-5	−4.1 (−9.5 to 13.6)	.14
>5	3.1 (−3.0 to 9.3)	.32
Self-reported health assessment		.26^b^
Good, very good, or excellent	1 [Reference]	NA
Fair	−2.3 (−7.5 to 2.9)	.38
Poor	−6.6 (−14.8 to 1.6)	.12

Abbreviations: CPR, cardiopulmonary resuscitation; GED, General Educational Development test; NA, not applicable.

^a^ Adjusted risk differences represent differences in predicted probability of preferring CPR compared with the referent category (adjusted for age, sex, race, ethnicity, highest educational level, spirituality/religiosity, time receiving dialysis, and self-reported health assessment). Predicted probabilities represent the expected proportion of patients in each category of exposure who would prefer CPR if all cohort members belonged to that category.

^b^ Omnibus *P* values.

### Association of Preference for CPR With Other Aspects of End-of-Life Care

Among the 84.2% (738 of 876) of participants who indicated that they would definitely or probably want CPR (CPR group), 555 (75.2%) participants vs 13 of 138 (9.4%) of those who did not want CPR (DNR group) wanted mechanical ventilation (*P* < .001). In the CPR group, 249 participants (33.7%) had documented their treatment preferences vs 84 of 138 participants (60.9%) in the DNR group (*P* < .001). With regard to values about future care, 171 participants (23.2%) in the CPR group vs 5 participants (3.6%) in the DNR group valued life prolongation (*P* < .001); 320 (43.4%) vs 109 of 138 (79.0%) (*P* < .001) valued comfort, and 247 (33.5%) vs 24 of 138 (17.4%) were unsure about their wishes for future care (*P* < .001). In the CPR group, 207 (28.0%) participants vs 62 of 138 (44.9%) in the DNR group had thought about stopping dialysis (*P* < .001), and 181 participants (24.5%) vs 58 of 138 (42.0%) had discussed stopping dialysis (*P* = .001). After accounting for multiple comparisons, no statistically significant associations were observed between CPR preference and documentation of a surrogate decision maker, thoughts or discussion of hospice, preferred place of death, expectations about prognosis, reported symptoms, or palliative care needs (Table 3).

**Table 3.** Association Between Participants’ Preference for CPR and Other Aspects of End-of-Life Care

End-of-life care domain	No. (%)			Adjusted risk difference, % (95% CI)^b^	*P* value
	Total (N = 876)	Resuscitation preference^a^			
		CPR (n = 738)	DNR (n = 138)		
Preference for mechanical ventilation	568 (64.8)	555 (75.2)	13 (9.4)	63.8 (57.3 to 70.2)	<.001
Advance care planning					
Documented surrogate	434 (49.5)	338 (45.8)	96 (69.6)	−13.5 (−22.7 to −4.4)	.004
Documented treatment preferences	333 (38.0)	249 (33.7)	84 (60.9)	−18.3 (−27.4 to −9.2)	<.001
Values about future care					<.001^c^
Longevity	176 (20.1)	171 (23.2)	5 (3.6)	17.7 (12.7 to 22.7)	<.001
Comfort	429 (49.0)	320 (43.4)	109 (79.0)	−33.0 (−41.2 to −24.8)	<.001
Unsure	271 (30.9)	247 (33.5)	24 (17.4)	15.3 (7.8 to 22.9)	<.001
Stopping dialysis					
Thoughts of stopping dialysis	269 (30.7)	207 (28.0)	62 (44.9)	−16.9 (−26.0 to −7.7)	<.001
Discussion of stopping dialysis	239 (27.3)	181 (24.5)	58 (42.0)	−15.7 (−24.7 to −6.8)	.001
Enrolling in hospice					
Thoughts of hospice	472 (53.9)	386 (52.3)	86 (62.3)	−7.0 (−16.0 to 1.9)	.12
Discussion of hospice	202 (23.1)	160 (21.7)	42 (30.4)	−6.1 (−14.2 to 1.9)	.14
Desired in-home death	519 (59.2)	427 (57.9)	92 (66.7)	−4.2 (−13.4 to 5.0)	.37
Expectation about prognosis, y					.04^c^
<5	104 (11.9)	71 (9.6)	33 (23.9)	−8.2 (−14.7 to −1.7)	.01
5-10	135 (15.4)	113 (15.3)	22 (15.9)	2.2 (−4.0 to 8.4)	.49
≥10	294 (33.6)	268 (36.3)	26 (18.8)	8.2 (−0.5 to 16.9)	.06
Unsure	343 (39.2)	286 (38.8)	57 (41.3)	−2.2 (−11.5 to 7.1)	.64
Reported symptoms					
Weakness or lack of energy	531 (60.6)	442 (59.9)	89 (64.5)	−1.1 (−9.9 to 7.7)	.81
Pain	456 (52.1)	390 (52.8)	66 (47.8)	2.5 (−6.7 to 11.6)	.60
Difficulty sleeping	443 (50.6)	377 (51.1)	66 (47.8)	1.9 (−7.4 to 11.2)	.69
Poor mobility	379 (43.3)	314 (42.5)	65 (47.1)	1.0 (−7.8 to 9.8)	.83
Anxiety	258 (29.5)	219 (29.7)	39 (28.3)	0.9 (−7.5 to 9.2)	.84
Shortness of breath	258 (29.5)	217 (29.4)	41 (29.7)	2.1 (−6.1 to 10.3)	.61
Depression	205 (23.4)	167 (22.6)	38 (27.5)	−6.2 (−14.3 to 1.9)	.13
Palliative care needs					
Discussion about future treatment options	426 (48.6)	365 (49.5)	61 (44.2)	1.7 (−7.6 to 10.9)	.73
Advance care planning	386 (44.1)	338 (45.8)	48 (34.8)	5.8 (−3.3 to 15.0)	.21
Discussion about care plan and treatment	282 (32.2)	241 (32.7)	41 (29.7)	−1.5 (−10.4 to 7.5)	.75
Help coping with sadness	243 (27.7)	204 (27.6)	39 (28.3)	−1.8 (−10.3 to 6.7)	.68
Discussion about finding meaning in life	151 (17.2)	134 (18.2)	17 (12.3)	2.6 (−4.6 to 9.8)	.48
Discussion about dying and death	112 (12.8)	98 (13.3)	14 (10.1)	2.4 (−3.7 to 8.4)	.45

Abbreviations: CPR, cardiopulmonary resuscitation; DNR, do not resuscitate.

^a^ Percentages by resuscitation preference are not adjusted for self-reported participant characteristics. These have been rounded and may not total 100.

^b^ Adjusted risk differences represent the difference between the predicted probability of each outcome if all cohort members selected CPR vs if all cohort members selected DNR (adjusted for age, sex, race, ethnicity, highest educational level, spirituality/religiosity, time receiving dialysis, and self-reported health assessment).

^c^ Omnibus *P* values.

## Discussion

Most individuals (84.2% [738 of 876]) receiving outpatient dialysis at nonprofit facilities in 2 US metropolitan areas who participated in our survey indicated they would definitely or probably want to be resuscitated if their heart were to stop beating. In analyses adjusted for self-reported participant characteristics, CPR preference was associated with some, but not all, of the other domains of end-of-life care that were examined. Furthermore, study participants’ CPR preferences were not always aligned with how they responded to questions about these other aspects of end-of-life care. These findings argue for caution in extrapolating patients’ values, preferences, knowledge, and expectations pertaining to other aspects of end-of-life care from their resuscitation choice. Stronger efforts are needed to improve education around CPR and contextualize discussions about resuscitation preference within a broader conversation about end-of-life wishes for members of this population.

In other populations, the percentage of patients expressing a preference for CPR have ranged from 22% to 93%, with marked differences depending on patients’ health state and illness severity.^32,33,34,35,36,37,38,39,40^ To our knowledge, only a few prior studies^19,20,21,41,42^ have asked people with advanced kidney disease about their resuscitation preferences. Although these studies were conducted in various clinical settings, geographic locations, and time periods and framed the question in different ways, a common finding across all studies was that a substantial number of participants wished to be resuscitated. Among the larger studies, the percentage of patients who preferred CPR has ranged from 39% of 584 Canadian patients with advanced kidney disease,^20^ to 65% of members of a sociodemographically diverse population of 423 patients receiving dialysis in Cleveland, Ohio,^21^ to 87% of 469 patients receiving maintenance dialysis in Kansas City, Missouri, Rochester, New York, and northern West Virginia.^19^ Consistent with the study by Moss et al,^19^ a preference for CPR among participants in the present study was associated with younger age and Black race. Although none of these studies explicitly examined the association between resuscitation preferences and responses to questions about other aspects of end-of-life care, most reported a high frequency of unmet palliative care needs and substantial symptom burden as well as poor knowledge and limited discussion about hospice. For example, Davison^20^ and Saeed et al^21^ reported that most patients surveyed wanted their nephrologists to be responsive to their spiritual, social, and psychological concerns. Davison^20^ also reported that, although 83% of participants thought it was very important to plan for end of life, less than half of study participants indicated they had discussed their end-of-life wishes with their care team. Saeed et al^21^ found that more than half (53%) of participants in their study preferred to die at home and almost 20% were unfamiliar with hospice. Our study adds to this earlier work by examining the association between resuscitation preferences and how patients responded to questions about other aspects of end-of-life care.

Although not required by the Centers for Medicare & Medicaid Services, preferences for resuscitation are often elicited as part of the dialysis consent process. How patients respond to questions about code status in this context has direct implications for how dialysis facility staff will react in the event of a cardiac arrest, although the results of a recent study^3^ suggested that approximately 81% of patients receiving care in a large dialysis organization who wanted to receive CPR were not resuscitated. Although there may be a place for a checklist approach to eliciting patients’ preferences about particular clinical interventions, especially when this choice pertains to risky procedures and/or crisis situations, this approach may have the unintended consequence of dislocating discussions about CPR from the broader process of advance care planning and bigger picture conversations about patients’ values and goals of care.^43,44,45^ A siloed approach to discussion of resuscitation preferences in this and other narrow contexts and/or crisis situations may result in missed opportunities to address misconceptions about CPR and explore apparent inconsistencies between patients’ resuscitation preferences and their values, preferences, knowledge, and expectations for future care. Although members of this study cohort who preferred CPR were more likely to value life extension than those who did not wish to be resuscitated, most valued relief of pain and discomfort or were unsure about their future care. Elicitation of resuscitation preferences without consideration of values and goals may lead to care that fails to support what matters most to each patient. We suspect that framing discussions about future care around patients’ values and goals rather than their preferences for specific interventions like CPR may be helpful in improving the quality of end-of-life care.^23,46,47,48^

Prior studies conducted in patients with kidney disease and in other populations support the feasibility and effectiveness of engaging patients and families in advance care planning.^42,49,50,51,52,53,54,55,56,57,58^ That a substantial proportion of participants herein who did not want to be resuscitated had documented neither a surrogate decision maker nor treatment preferences illuminates an important gap in care. A default approach to resuscitation in many health care systems makes these patients especially vulnerable to receiving care that is incongruent with their preferences.^59^ Furthermore, 1 in 4 patients in the present study who wanted to receive CPR did not want to receive mechanical ventilation, highlighting the importance of efforts to educate patients and families about what CPR typically involves, the context in which this intervention tends to be delivered, and the most likely outcomes. Such efforts should focus not only on the low likelihood of surviving an episode of CPR for members of this population but also on the clinical context, and specifically the tendency of successful CPR to serve as a gateway to other intensive interventions intended to prolong life, such as mechanical ventilation and intensive care unit admission.^60,61^ Prior research in other populations suggests that patients are less likely to want to be resuscitated when they are informed about the expected outcome.^32,62,63,64^

Although palliative care specialists are often called on to support discussions of goals of care and treatment preferences, their training also equips them to address patients’ palliative care needs and symptom burden. Palliative care consultation has been associated with improvement in symptom burden in other populations^36,65^ but is underused for patients receiving maintenance dialysis.^15,66^ Although little is known about the feasibility, acceptability, and effectiveness of enhancing palliative care support for patients receiving maintenance dialysis,^52,55,67^ the very high prevalence of palliative care needs and symptom burden among these individuals highlights the potential value of efforts to improve their access to palliative care.^58,66,68,69,70,71,72,73,74^

### Limitations

Our study has several important limitations. First, the results of this cross-sectional survey study conducted at nonprofit dialysis facilities in 2 US metropolitan areas may not be generalizable to patients receiving dialysis at for-profit facilities, those living in other parts of the country, and those who were excluded from our study (non–English-speaking individuals and persons unable to provide written informed consent). Although the demographic characteristics of our cohort resemble those of the overall US population receiving dialysis,^24^ the high percentage of patients enrolled in our study who rated their health as good, very good, or excellent could mean that their CPR preferences are not generalizable to the overall US population undergoing dialysis. Second, although we used an existing instrument to elicit participants’ CPR preferences,^32^ our approach has some inherent limitations and provides only a partial understanding of these preferences. For example, a Likert-type scale is subject to response bias,^75^ and the scale we used did not include neutral or uncertain response options. We also did not frame the question about CPR preference with background information about the intervention and expected outcomes. Nor did we ask about patients’ resuscitation preferences in different hypothetical future health states, elicit trade-offs, assess for stability in preferences over time, or collect information on dialysis facility resuscitation policies. Third, how individuals responded to survey items may differ from responses that they might provide during discussions with clinicians about goals of care because of differences in context.

## Conclusions

Most patients undergoing dialysis at 31 nonprofit facilities in 2 US metropolitan areas who participated in this study indicated they definitely or probably wanted to be resuscitated if their heart were to stop beating. Resuscitation preferences were associated with responses to some, but not all, questions about other domains of end-of-life care. Furthermore, participants’ CPR preferences were not always aligned with how they responded to questions about these other aspects of end-of-life care. These findings underline the importance of educational initiatives to improve patients’ understanding of the clinical context and outcomes of CPR and of contextualizing discussions about code status in a broader conversation and understanding what matters most to each patient.

## Notes

Supplement. **eAppendix.** United States Renal Data System Study of Treatment Preferences (USTATE) Patient Questionnaire
