# Errors in the Number of Patients and Results

**DOI:** 10.1001/jamanetworkopen.2021.15787

**Published:** 2021-06-14

In the Original Investigation titled “Cardiopulmonary Resuscitation Preferences of People Receiving Dialysis,”^1^ published August 24, 2020, 2 surveys from 3 study participants were erroneously included in the analyses. After excluding the results of the second survey for each of these 3 participants, 1592 patients were approached, 1431 were invited to participate, 1006 agreed to participate, and 873 (61.0%) were included in the corrected analyses. The corrected analyses resulted in changes to numerators, denominators, percentages, and effect sizes throughout the text and tables; most results differ from the original article by less than 1 decimal point. In addition, the omnibus *P* value for the association of time on dialysis with preference for cardiopulmonary resuscitation did not reach the threshold for statistical significance in the corrected analyses; the correct *P* value is .052. Thus, self-reported participant preference for cardiopulmonary resuscitation was not associated with receiving dialysis for longer periods. This article has been corrected.^1^ In addition, 1 number was corrected in the related Invited Commentary.^2^
